# Wildfire Selectivity for Land Cover Type: Does Size Matter?

**DOI:** 10.1371/journal.pone.0084760

**Published:** 2014-01-13

**Authors:** Ana M. G. Barros, José M. C. Pereira

**Affiliations:** Centro de Estudos Florestais, Instituto Superior de Agronomia, Universidade de Lisboa, Lisboa, Portugal; The Ohio State University, United States of America

## Abstract

Previous research has shown that fires burn certain land cover types disproportionally to their abundance. We used quantile regression to study land cover proneness to fire as a function of fire size, under the hypothesis that they are inversely related, for all land cover types. Using five years of fire perimeters, we estimated conditional quantile functions for lower (avoidance) and upper (preference) quantiles of fire selectivity for five land cover types - annual crops, evergreen oak woodlands, eucalypt forests, pine forests and shrublands. The slope of significant regression quantiles describes the rate of change in fire selectivity (avoidance or preference) as a function of fire size. We used Monte-Carlo methods to randomly permutate fires in order to obtain a distribution of fire selectivity due to chance. This distribution was used to test the null hypotheses that 1) mean fire selectivity does not differ from that obtained by randomly relocating observed fire perimeters; 2) that land cover proneness to fire does not vary with fire size. Our results show that land cover proneness to fire is higher for shrublands and pine forests than for annual crops and evergreen oak woodlands. As fire size increases, selectivity decreases for all land cover types tested. Moreover, the rate of change in selectivity with fire size is higher for preference than for avoidance. Comparison between observed and randomized data led us to reject both null hypotheses tested (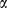 = 0.05) and to conclude it is very unlikely the observed values of fire selectivity and change in selectivity with fire size are due to chance.

## Introduction

Wildfire is a ubiquitous disturbance in many ecosystems of the world [1], [2]. Unlike other ecological disturbances, such as cyclones or earthquakes, fires feed on complex organic molecules transforming them into organic and mineral products, thus acting as an evolutionary force that shapes fire-prone ecosystems and plays a pivotal role in maintaining their structure and function [3].

What is common to any fire-prone ecosystem is that its fire regime - broadly described in terms of fire occurrence, spread, behavior and effects - results from non-linear processes controlled by the interactions and feedbacks between fire, land use, vegetation attributes, climate, landscape characteristics and ignition patterns. Understanding the mechanisms that determine where and when fires occur, what limits their growth and intensity, and what will be the ecosystem response is an essential goal of fire ecology and management [4]–[6]. Past studies addressed the relationships between climate, fuel, topography, in what is commonly described as the fire behaviour triangle [4], [7]–[10]. Over the past years, in Southern California and in the Mediterranean Basin regions, the relative contribution of fuel 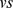 climate has been the focus of attention and remains an issue under debate, with strong arguments on both sides and important management implications [8], [11]–[14]. If, in fact, fuels (age, load and continuity) are determinant for the occurrence of severe fire seasons, then fuel management may be efficient in reducing fire hazard [15]. However, if area burned tends to coincide with episodes of severe weather, poorly constrained by fuel landscape properties, then investments in large scale fuel management programs are unlikely to succeed [16], [17]. Furthermore, future climate change is likely to exert stronger impact in fire regimes that are primarily climate-driven. The relative dominance of fire regime controls can also change in time. A recent study [18] identified a major shift in the fire regime of the Western Mediterranean Basin over the past 130 years, with a change point circa 1970. In the decades after 1970, number of fires doubled and burned area increased by one order of magnitude. The authors show that the main driver of this shift was the increase in fuel amount due to rural abandonment. Additionally, it was shown that climatic conditions were poorly related to pre-1970s fires and strongly related to post-1970s fires, suggesting that fires are currently less fuel-limited and more drought-driven than before the 1970s[18]. Similar results were found by [19] for the eastern Mediterranean region using historical fire data between 1894 and 2010. Trend analysis of number of fires and air temperature showed a statistically significant increase, particularly after the mid-1970s, and strong correlations between fire occurrence (number of fires and burnt area) and mean maximum and the absolute maximum air temperature [19].

The landscape in the Mediterranean basin is highly dynamic, humanized and fire-prone, therefore land use/land cover changes (LULC) have strong effects on fire hazard through changes in vegetation structure, fuel load, and fuel composition [12], [20]–[22]. A better understanding of the relationship between fire and fuel provides the basis for analyses that attempt to quantify land cover proneness to fire or, alternatively, fire selectivity towards different land cover types. The concept of fire selectivity, also known as fire selection ratio, is derived from seminal work of [3], whom used the metaphor of fire as global herbivore capable of significantly alter biomass in flammable ecosystems [3]. The interpretation of fire selectivity is straightforward - fires are considered selective if they burn through a certain land cover type disproportionally to its availability. Selectivity is positive (preference) if the land cover is burned proportionally more than available and is negative (avoidance) if the land cover class is burned proportionally less than available. When a land cover type is consumed proportionally to its availability the fire is considered indifferent to that land cover type.

Previous analyses of fire selectivity towards different land cover types in Portugal suggest that shrublands and conifer stands are more fire prone, while agricultural areas are less fire prone [20], [23]–[26]. Within forest types [27] found that the most fire prone forest type is maritime pine, followed by eucalypt and unspecified broadleaf species, while stone pine is the least fire prone. Land cover proneness to fire and terrain was analyzed by [28]. In agreement with other analyses, shrublands and steep slopes were found to be the most fire prone land cover/topographic setting. Broad scale analysis of fire selectivity to land cover and topography in southern European countries suggested that shrublands and grasslands were preferred by fire, with forests showing intermediate values of selectivity. Results also show that north-facing slopes steeper than 25% were less susceptible to burning [29]. In a study for Sardignia, Italy, [30] showed that the mean fire size of grasslands and shrublands is significantly larger than expected using a random null model, whereas in urban areas and permanent crops there is significant resistance to fire spread. In central Spain, and based on data from 16 years, [31] concluded that pine woodlands showed significant and positive fire selectivity, whereas deciduous woodlands showed significant and negative selectivity. Their study also shows that fires positively selected areas closer to towns and roads, and that selectivity to topographic variables - slope and aspect - was less marked than for land cover and proximity to towns and roads [31].

Despite overall agreement between fire selectivity analyses, their conclusions may be hindered by the effects of other drivers of fire spread, e.g. fuel connectivity, structure and load, landform position, level of suppression and weather conditions, which can overwhelm the importance of land cover type [24], [32]. Furthermore, [31] elaborates on the shortcomings associated with nature of fire data and fire selectivity analyses that may affect the findings of some studies. The authors propose assessing fire selectivity through a null based model based on fire size and shape that is independent of spatial relationships or any other distributional bias of fire data [30], [31]. Under severe weather conditions fires are expected to become larger and, to a certain extent, independent of land cover patterns [4], [33], [34], i.e. it is likely that as fire size increases, selectivity becomes less important, as other drivers, e.g. weather, become increasingly important [4]. The effect of fire size on fire selectivity was addressed by [26], whom analyzed selectivity in distinct fire size classes. The authors concluded that large (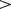
5 km^2^ and 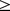
15 km^2^) and very large (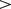
15 km^2^) fires are not significantly selective for land cover, while small fires (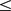
5 km^2^) are unequivocally selective. [31] also examined the size-dependent variation in the degree of fire selectivity, by dividing fires into small (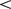
100 ha) and large (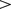
100 ha) and found no differences in the land cover selection indexes between both groups.

The present study aims to discern to what extent fire selectivity towards different land cover varies as a function of fire size. We used quantile regression to determine how selectivity responds to increasing fire size, for different land cover types. Quantile regression allows studying the edges of the response variable distribution (selectivity), conditioned on values of the predictor variable (fire size). In this context, for any given land cover type lower quantiles correspond to avoidance, while higher quantiles correspond to preference for that specific land cover. Under the assumption that land cover types are differently prone to fire, we expect to find different rates of change in both upper and lower quantiles, among different land cover types. These rates of change in selectivity will be quantified by the slope of the estimated linear regression quantiles and compared with a null random model, to test the hypotheses that land cover proneness to fire is independent from fire size. The present study is meant to contribute to the current debate on the importance of weather 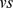 fuel as determinants of fire regime attributes [35], [36], with implications for the land use planning and fuel management in the wildland-urban interface. From the fire management stand-point, in land cover types with higher rates of change in selectivity escalating fire behavior is likely to be quicker, thus with potential for severe fire behavior. Based on that knowledge, more accurate assessment of potential fuel management practices and improved fire suppression strategies can be developed.

## Data and Methods

### Study area and data

**Figure 1:**
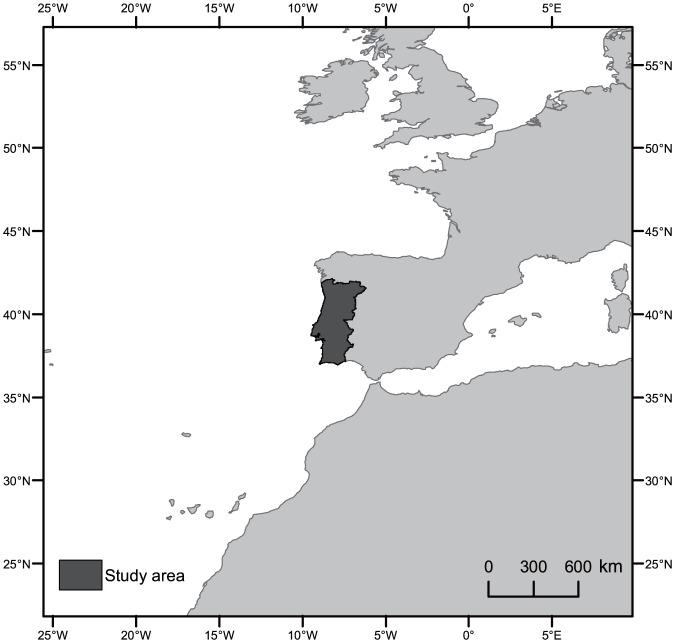
Study area corresponds to mainland Portugal located in the Iberian Peninsula, Europe.

The study area, with approximately 89,100 km^2^, corresponds to mainland Portugal, located in south-western Europe (Figure 1). The Portuguese climate is temperate, with lower mean annual temperature along the coast and in the northern half of the country [37]. Precipitation is higher in the interior highlands of northern Portugal than in the southern half of the country. The dry season, corresponding to about 6% of the mean annual precipitation, is concurrent with the fire season and takes place during the summer months, usually June to September [37]. According to the latest revision of the National Forest Inventory (2010), forested lands (plantations or woodlands) are the most common land cover type, representing 40% of Portugal's mainland surface area. From the latter, 27% corresponds to maritime pine stands (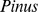 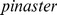), 23% to blue gum stands (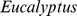 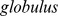) and 23% to cork oak (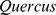 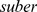). Maritime pine stands occur mainly in the northern half of the country, while blue gum stands are predominant in the western half, central and southern of Portugal. Evergreen oak woodlands of cork oak and holm oak (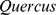 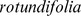) are abundant in the south-western and south-eastern portions of the country, respectively. Agriculture covers one third of the Portuguese mainland and is widely present throughout the study area, particularly in the central costal plain and along main river valleys and alluvial plains. Shrublands represent 22% of Portugal's surface and are primarily found in eastern half of the country and/or in mountainous regions with low population density.

Wildfire is the most influential process shaping short-term landscape dynamics in the study area [24], [38]. Portuguese pyro-geography is quite heterogeneous in terms of fire ignitions and burned areas. Ignitions are spatially concentrated and more frequent in coastal and peri-urban areas, but due to higher population density, accessibility and landscape fragmentation, seldom originate large burned areas [39]. Large fires, responsible for the vast majority of the burned area occur in the northern interior part of the study area, where lower demographic pressure coupled with higher fuel connectivity and fuel loads contribute to occurrence of most extensive burning [20]. The majority of area burned (80%) in Portugal is due to fire events occurring during a small number (10%) of summer days, when the typical atmospheric circulation pattern is characterized by an upper level ridge located over the Iberian Peninsula and conducive to severe fire weather [40], [41].

**Figure 2:**
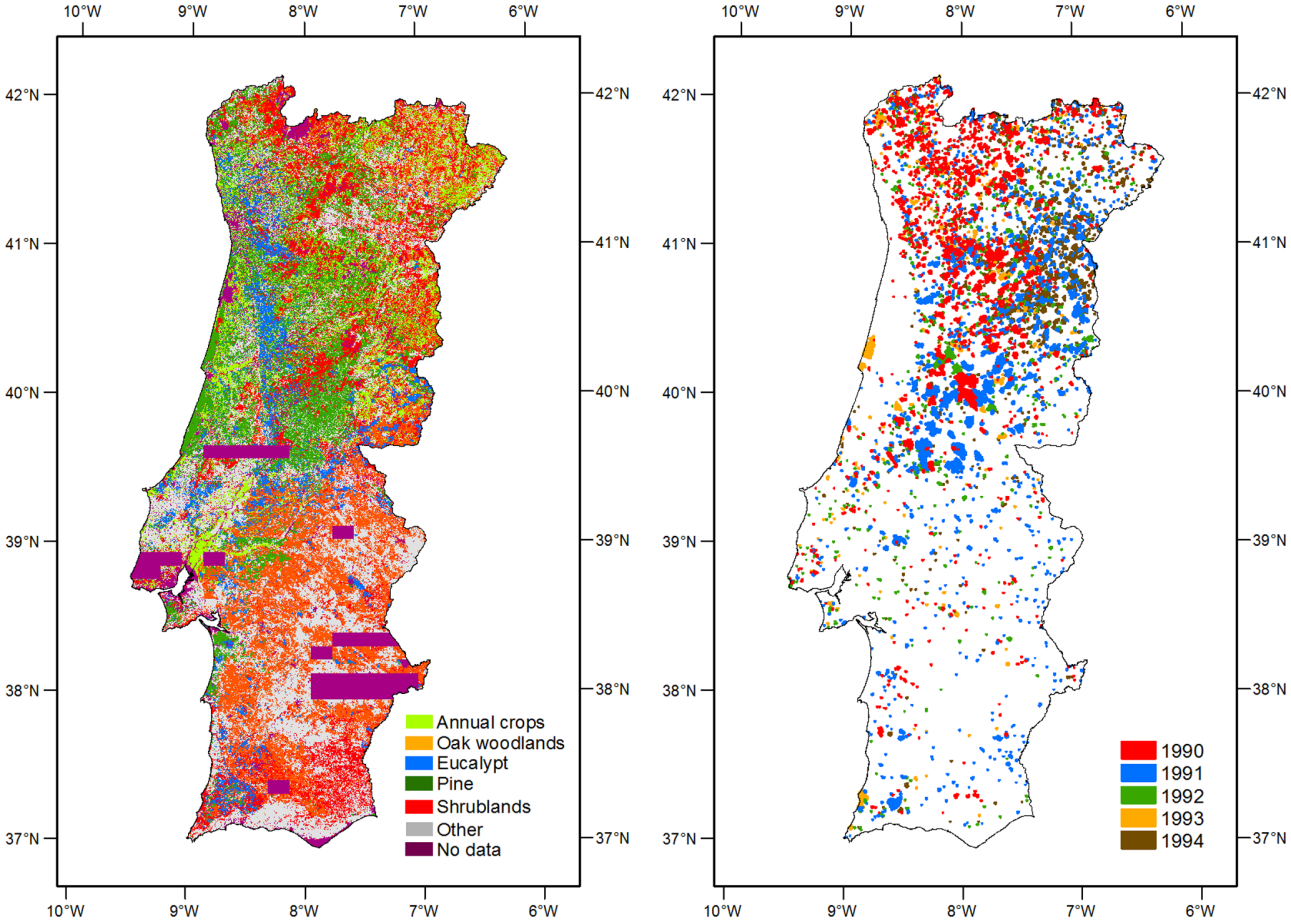
Land cover map (right) and spatial distribution of fire perimeters (left). The land cover map is based on a simplification of the COS90 legend. Annual crops correspond to 10% of the study area, whereas evergreen oak woodlands, account for 18% of the study area. Forests are mostly 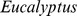 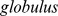 plantations (Eucalypt, 6%) and 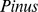 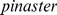 stands (Pine, 15%). Shrublands and grasslands account for 16% of the study area. The fire perimeter map represents the annual fire perimeter distribution per year between 1990–1994. Areas that burned twice during the study period account for 2.5% of the overall burned area.

The Portuguese fire atlas, derived from satellite imagery, includes fire perimeters from 1975 to 2009 [42]. Annual fire perimeter maps were obtained with semi-automatic supervised classification of single-date, post-fire season Landsat imagery, performed with the Classification and Regression Trees (CART) algorithm of [43]. We relied primarily on near-infrared and mid-infrared channels, and thus were able to avoid atmospheric correction of the data. In each year, we performed relative radiometric calibration of the eight Landsat images required to form the mosaic covering the entire mainland of Portugal, ensuring consistent performance of the image classification rules induced with CART. Accuracy of the data was assessed by comparing mapped burned area with field statistics gathered on the ground by the National Forest Authority, and by the National Civil Protection Authority, for over 3000 administrative units. The Portuguese fire perimeter database can be accessed at www.icnf.pt. We used fire data from the 1990–1994 fire seasons, with spatial resolution of 30 m and minimum mapping unit of 5 ha (Figure 2). In the five years considered in this study, a total of 5,712 fire perimeters were mapped (Table 1). Overall burned area over this period was 442,924 ha, with mean fire size of 77 ha (Table 1).

**Table 1:** Number of fires and burned area per year.

Year	Number of fires	Burned area (ha)
1990	1670	113,136
1991	1552	161,205
1992	712	34,219
1993	467	45,447
1994	1311	88,917

During the study period, 1990–1994, total number of fires and burnt area corresponds to 5,712 and 442,924, respectively.

To characterize land cover in the study area we used the 1990 land cover map (COS90) [44]. COS90 is a land cover classification map for mainland Portugal, derived from aerial photography collected between June and September of 1990 (Figure 2). It has a minimum mapping unit of 1 ha and is available in vector format at www.igeo.pt. Similar to other studies that have addressed relationships between land cover and wildfire in Portugal [20], [23], [26]–[28], the choice of time frame for the analysis was opportunistic, due to limited land cover data availability at an adequate spatial resolution for the objectives of this study. The original COS90 legend, which is hierarchical, with the most detailed level containing 76 classes, was simplified to include the following classes: annual crops, evergreen oak woodlands, eucalypt stands, maritime pine stands and shrublands/grasslands (Figure 2). Over the study period, a small proportion (2.5%) of the burned are was burned twice. Using the burned area maps of 1990–1993, we reclassified all burned areas to shrublands for analysis in the following year, with exception of annual crops, which maintained the same class after the fire [27], [45].

### Selectivity index: Jacobs' index

Analysis of resource selection often relies on selection indices that summarize information on resource use and availability - see [46] for an extensive review of commonly used selectivity indexes. In this study we follow a type III design, where used and available resources are defined for each fire observation. The definition of available resources is a key component in resource selection analyses because it will determine the amount and accessibility of what is available and it is strongly affected by scale [47]. Resource selectivity was described by [48] as a hierarchical system of choices in which higher level choices condition lower level choices - e.g. the definition of an animal's home range will determine the amount of resources that will be available to it [48]. In this system, resources consumed can only be determined after defining resources available [47]. The analogy can be adapted to fire selectivity, where the spatial location of an ignition is the higher order process that conditions the type, amount and accessibility of land cover available, once that ignition becomes a spreading fire.

**Figure 3:**
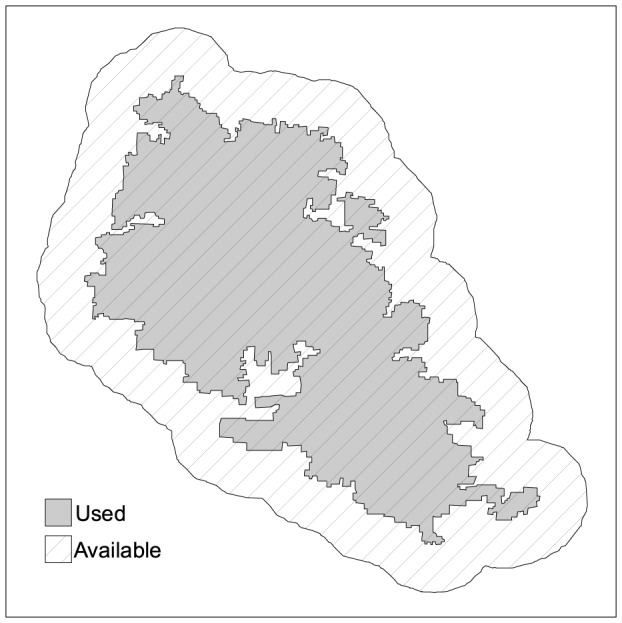
Example of the delineation of used and available area. The used area corresponds to the area actually burned by the fire (grey). The available area (dashed) corresponds to the used area plus the area of a surrounding buffer (white). The buffer is delineated to have the size of the original fire, therefore the available area is twice the used (burned) area.

We define available area as twice the used area, by delineating a buffer around each fire perimeter with the same area as the fire [26], [29]. We define used area as the area burned by the fire (Figure 3). Our choice is justified in two ways: 1) buffer size is the same size of the fire, so that sampling effort is equivalent for both and 2) used area (fire size) is included in the available area because, by definition, used resources are a subset of available resources [47].

The use of selectivity ratios to assess differential degree of burning of land cover types fire is common in the literature [20], [23], [26]–[28], [30], [31], [49]. Most studies have used Savage's forage ratio as a selectivity index [46]. The ratio theoretically varies between 0 (avoidance) and 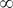 (preference), taking the value of 1 when proportion used equals proportion available - no preference or avoidance by fire 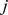 of land cover type 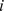. It follows that the forage ratio will present a larger region for preference than for avoidance, which may introduce bias in the statistical analysis. To minimize such potential bias we chose to use Jacobs' index, which has a bounded scale between -1 and 1 and is symmetric around 0 (indifference). For fire 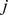 and resource 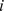, Jacobs' selectivity index is defined as [50]:
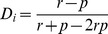
(1)where 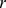 is the proportion of land cover type 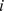 used by fire 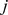 and 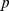 is the proportion of land cover type 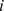 available to fire 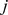. Jacobs' index ranges between -1 (extreme avoidance) and 1 (extreme preference), taking the value 0 under indifference [51], [52].

In this study, each observation consists of a fire (resource used) and its buffer area (resource available). For each observation, a value of the index was calculated for each land cover type contained within the fire+buffer boundaries. Therefore, the number of observations in each land cover class will depend on the number of fire perimeters (or buffers) intersecting each class. Averaging fire selectivity over all fires allows for inference regarding the fire proneness of a specific land cover class.

### Linear quantile regression and randomization tests

Quantile regression is used to estimate and draw inferences about conditional quantile functions [53], [54]. A regression quantile of 0.10 will estimate a function of the predictor variables, such that 10% of the observations are below it, while a regression quantile of 0.5 (median) splits the frequency distribution into two parts, each containing equal number of observations [55], [56]. Quantile regression is particularly useful when one is interested in modeling the effect of covariates along or near the upper boundary of the conditional distribution of responses, instead of the mean response modeled by common statistical techniques, such as ordinary least squares. An excellent primer on quantile regression for ecologists can be found in [56].

We used linear quantile regression to examine the relationship between fire size and fire proneness for different land cover types. Quantile regression is particularly useful in this case because the edges of the selectivity index distribution have distinctive interpretations. For any given land cover class, lower quantiles represent fires that burn through a specific land cover proportionally less than available (lower end of the distribution - avoidance region), while upper quantiles represent fires that burn proportionally more than available (upper end of the distribution - preference region). Therefore, we are interested in understanding how the predictor (fire size) affects the distribution in these particular regions of the response variable distribution, rather than the mean response of fire selectivity to changes in fire size. Additionally, quantile regression analysis does not require 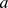 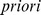 segmentation of fire observations according to size[26], [31]. Steepness of the slope of each quantile regression line quantifies the rate of change in fire selectivity as fire size increases.

We computed and plotted estimates of linear quantile regression functions for the first significant upper and lower quantiles of fire selectivity. To deal with clumped discrete values of -1 and 1 we jittered these values by adding 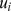, which is i.i.d. U[0,0.01], thus replacing discrete values of -1 and 1 with a smoothed response [57]. Identification of significant quantiles was done in increments of 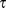 = 10 and significance was tested for 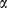  = 0.05. We present parameter estimates and their respective p-value (95% confidence level). All calculations and plots were performed in R, using the 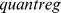 package [58].

To test whether fire selectivity and changes in selectivity as function of fire size differ from random we tested the observed values of fire selectivity and quantile regression slope against a null model of randomly placed fires. The objective of the null model is twofold: 1) to test whether observed selectivity and fire-size dependencies are not due to an artifact of sampling design, in which large fires are larger sampling units; 2) assess the impact of landscape configuration in the observed patterns of fire selectivity - small fires are more likely to occur within a single land cover type than larger fires, thus are more likely to exhibit strong selectivity - preference or avoidance [31].

**Figure 4:**
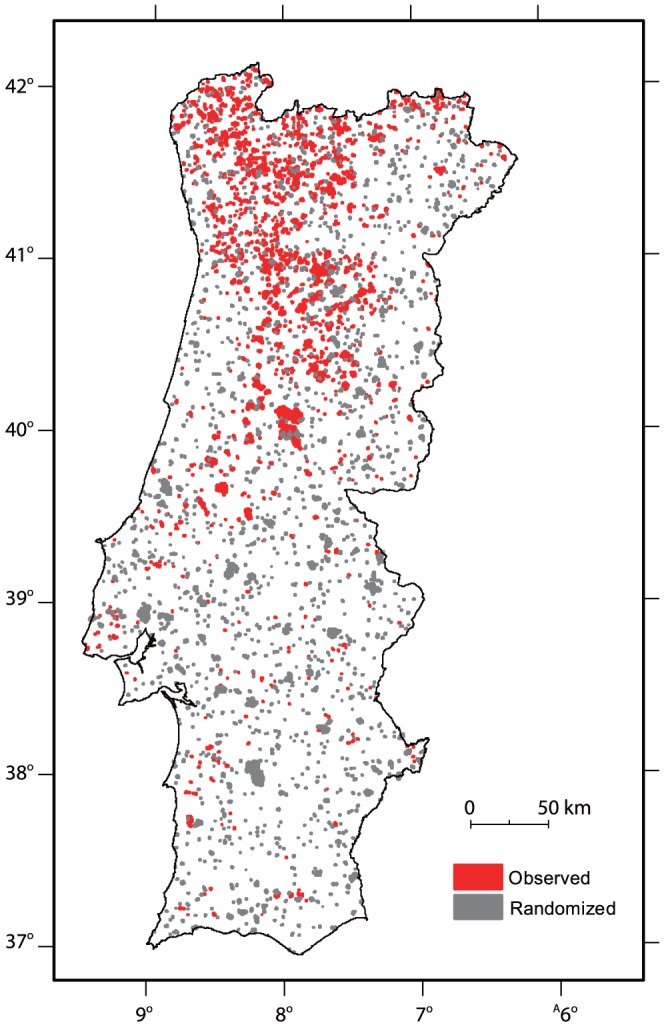
Example of one of the 500 maps showing the distribution of randomly placed fire perimeters (grey) overlapping observed fire perimeters (red). Each observation corresponds to a fire and its associated buffer, which is subjected to a random translation and rotation.

To test the null hypothesis that fire selectivity does not differ from selectivity obtained from a spatially random distribution of fires, we randomly re-positioned fire observations from the 1990 fire season. This corresponded to 1670 fire perimeters, which where rotated and translated, to obtain 500 maps of randomly distributed fire perimeters with the same shape and size distribution as the original data (Figure 4). We assumed that fire is selective to a given land cover type if its observed mean selectivity (average of Jacobs' ratio for all observations in that land cover type) was greater/smaller than 5% of 500 random fire distributions (one-sided test). P-values are calculated as the ranking position of the observed value in the simulated distribution. The same procedure was followed to test the null hypothesis that rate of change in fire proneness (slope in estimated upper/lower quantiles) does not differ from that which would be expected by chance alone.

## Results

**Figure 5:**
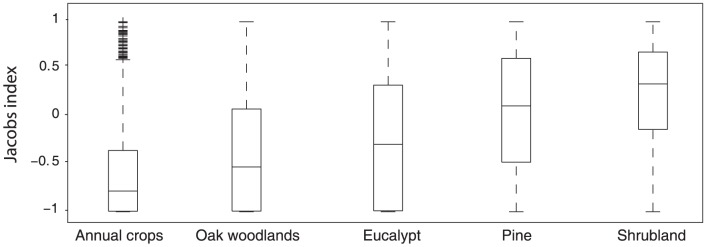
Jacobs' selection index with values of 0, 1 and −1 corresponding to indifference, preference and avoidance, respectively. Boxplot represents the 25th percentile (lower end of box) and 75th percentile (upper end of box). The median (50th percentile) is represented by the bar inside the box. Whiskers represent extreme observations and horizontal lines represent outliers - an observation is considered outlier if it is larger than q3
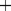
w(q3
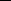
q1) or smaller than q1
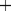
w(q3
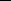
q1), where w, q1 and q3 are the whisker length, the 25th and 75th percentiles, respectively.

Results showed that fires exhibit different degrees of selectivity towards the various land cover types (Figure 5). Median fire selectivity showed that annual crops, evergreen oak woodlands and eucalypt plantations tend to be avoided by fire, while pine stands and shrublands tend to be preferred (Figure 5). The ranking of land cover types according to fire proneness, from less to most fire prone is: annual crops (−0.78), evergreen oak woodlands (−0.38), eucalypt plantations (−0.53), pine stands (0.11) and shrublands (0.35). This is in agreement with other studies that obtained similar rankings using Savage's forage ratio [20], [23], [26], [27].

**Figure 6:**
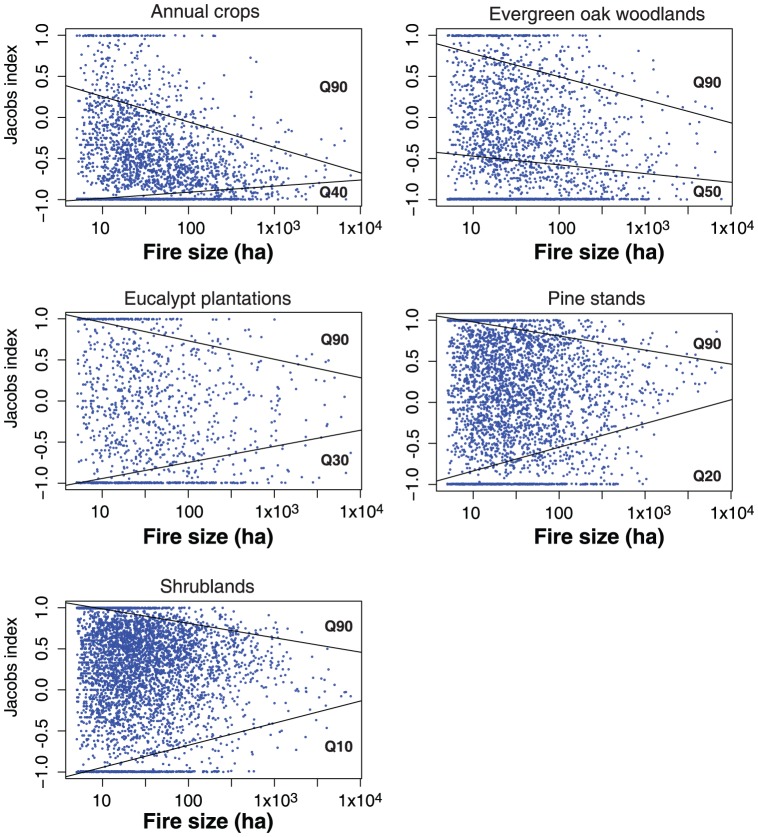
Quantile regression for the first significant upper and lower quantiles (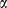  = 0.05) between fire size and fire selectivity (Jacobs' index). Significant quantiles in the avoidance region (lower) varied between 50th and 10th quantile in evergreen oak woodlands and shrublands, respectively. In the preference region all land cover types presented significant regressions for the 90th quantile. Estimated parameters and significance for each model are presented in Table 2.

All land cover types exhibit reductions in fire preference (upper quantile) as fire size increases (Figure 6). Slope values were negative and significant (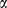 = 0.05) for the 90th quantile in all land cover types. Annual crops and pine stands showed the highest and lowest rate of change (decrease) in fire preference with fire size, as described by the slope of the estimated quantile regression, −0.31 and −0.17, respectively (Table 2).

**Table 2:** Linear regression coefficients for the estimated quantile functions.

Land cover	Parameter	Value	S.E.	t-value	p-value
Annual crops — 40th	Intercept	−1.059	0.015	−68.116	0.000
	Slope	0.074	0.014	5.151	0.000
Annual crops — 90th	Intercept	0.564	0.070	8.0691	0.000
	Slope	−0.309	0.340	−9.107	0.000
Evergreen oak woodlands — 50th	Intercept	−0.365	0.008	−5.400	0.000
	Slope	−0.106	0.036	−2.927	0.003
Evergreen oak woodlands — 90th	Intercept	1.056	0.085	12.316	0.000
	Slope	−0.280	0.046	−6.042	0.000
Eucalypt plantations — 30th	Intercept	−1.139	0.076	−13.764	0.000
	Slope	0.196	0.051	3.821	0.000
Eucalypt plantations — 90th	Intercept	1.180	0.066	18.123	0.000
	Slope	−0.220	0.042	−5.378	0.000
Pine stands — 20th	Intercept	−1.124	0.075	−14.890	0.000
	Slope	0.288	0.043	−6.73	0.000
Pine stands — 90th	Intercept	1.149	0.019	60.709	0.000
	Slope	−0.171	0.016	−10.597	0.000
Shrublands — 10th	Intercept	−1.211	0.069	−17.475	0.000
	Slope	0.269	0.052	5.148	0.000
Shrublands — 90th	Intercept	1.160	0.013	88.175	0.000
	Slope	−0.175	0.111	−15.774	0.000

For each land cover type we present the first significant upper and lower quantiles (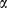 = 0.05). Also reported are the results of the t-test for assessing the significance of the regression parameters (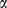 = 0.05).

Figure 6 shows, for each land cover type, the first significant quantiles in the avoidance (lower) and preference (upper) regions. It is worth mention that significant quantiles associated with fire avoidance varied according to the land cover type tested - the first significant quantile for evergreen oak woodlands was the median (50th quantile) while for annual crops it was the 40th quantile. Eucalypt plantations, pine stands and shrublands had significant regressions at the 30th, 20th and 10th quantiles, respectively. On the other hand, in the preference region, all land cover types presented significant regressions for the 90th quantile (Figure 6).

With the exception of evergreen oak woodlands, all land cover types exhibit reductions in avoidance as fire size increases (as shown by the positive slope values for estimated regression in the lower quantiles, Table 2). Rate of change in avoidance is positive and similar in pine stands and shrublands, 0.288 and 0.269, respectively. Decrease in avoidance for annual crops is the lowest of all, with a slope of 0.074. Evergreen oak woodlands displayed negative slope, −0.11), suggesting increasing avoidance with fire size (Table 2).

**Figure 7:**
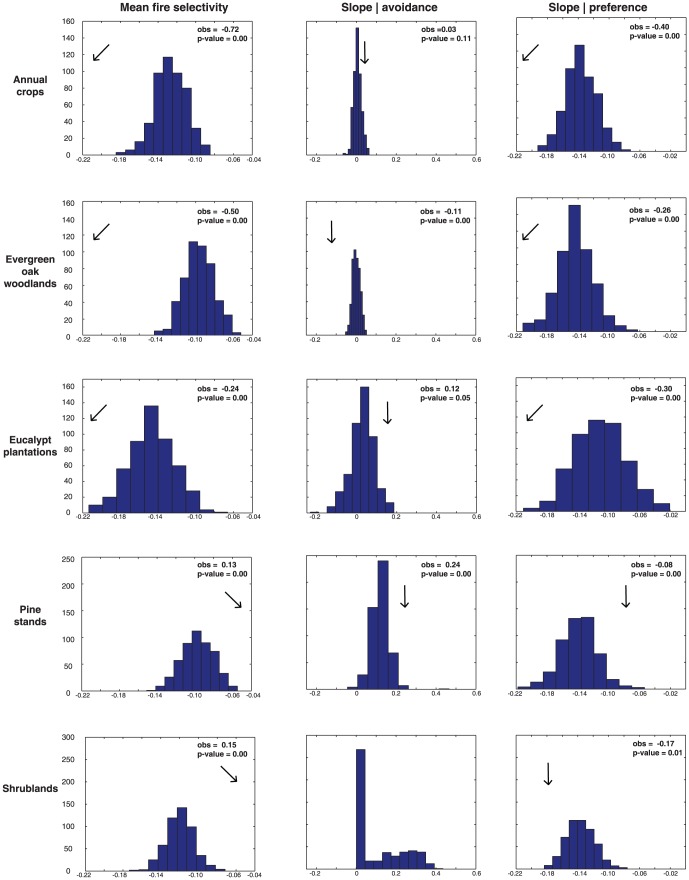
Expected distribution of the mean value of Jacobs' index and slope associated with significant (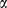  = 0.05) lower/upper linear regression quantiles after randomly re-assorting fire perimeters 500 times. The histograms have common scale in the x-axis for all land cover types and the same scale in the y-axis for the three variables - mean fire selectivity and slope of avoidance/preference regressions. Arrows indicate the position of the real (observed) p-value in the randomized distribution. P-value is calculated as the ranking position of the observed value in the simulated distribution.

Comparison between the randomly placed fire perimeter dataset and actual locations led to the rejection of the null hypothesis (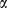  = 0.05) that mean fire selectivity does not differ from mean fire selectivity obtained through a spatially random distribution of fires (Figure 7). The relationship between fire selectivity and fire size was tested through two distinct null hypotheses that compare slope of lower/upper quantiles in observed and randomized data. For all land cover types tested, we rejected the null hypothesis that slope in avoidance quantiles does not differ from that obtained for the same quantiles with a randomized dataset (Figure 7). This hypothesis was not tested for shrubs because all quantiles below the median in the 1990 fire data were non-significant (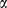  = 0.05). With the exception of eucalypt plantations (p-value  = 0.06), we also rejected the null hypothesis that slope in preference quantiles does not differ from slope obtained with randomized data (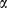  = 0.05, Figure 7).

## Discussion

According to this analysis, rates of change in selectivity as a function of fire size differ for preference and avoidance. For all land cover types tested, with exception of preference in eucalypt plantations (p-value = 0.06), we reject the null hypothesis (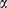 = 0.05). This suggests that for all other land cover types, the observed values of fire selectivity are significantly different from those that would be observed if fires were to occur at random locations in the landscape. Likewise, as fire size increases, changes in fire selectivity occur with magnitude (slope of the quantile regression estimates) that is significantly different from what would be observed due to chance alone. This suggests that change in selectivity as function of fire size, is in fact, a property of fire selectivity, and not a statistical artifact due to increase of sampling unit size in large fires. This is partially in agreement with previous research, since [26] found differences in fire selectivity with fire size, while [31] did not. However, comparison between both studies and with our study should be done with caution, because both [26] and [31] used discrete and distinct fire size thresholds to distinguish between small, large and very large fires.

Results showed that fire selectivity is generally higher for shrublands, pine stands and eucalypt plantations than for annual crops and agroforestry lands. This agrees with previous studies that identified patterns of strong fire preference for shrublands [26]. In a similar analysis, using Savage's forage ratio and the 1990–1994 fire perimeter database,[23] found that shrublands were the only land cover that burned more than expected. In their analysis, forests (both conifer and broadleaved) ranked second, with crops and agroforestry systems being the land covers least preferred by fire.

In general, all land cover types showed changes in fire preference (90th quantile) as fire size increases. Such changes corresponded to reductions in selectivity towards indifference (Jacobs' index  = 0), hence the negative slope estimated for the linear quantile regression at 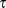 = 0.90 (significant at 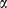 = 0.5). Analysis of quantile regression in the avoidance region (lower quantiles of fire selectivity distribution) showed more diverse results. Significant quantiles vary according to land cover types from 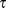 = 0.5 in evergreen oak woodlands, to 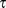 = 0.10 in shrublands. In general, we observed that as selectivity increases, the lower the first significant quantile. This may be due to the fact that, on average, land cover types with lower selectivity (annual crops, evergreen oak woodlands) have a significant number of fire observations with Jacobs' index equal to −1. This corresponds to situations where the land cover in question was available but not used, representing perfect avoidance. When data are populated with a significant number of low value observations, then it becomes necessary to increase 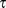 to obtain significant changes in fire selectivity as fire size increases (Figure 6). Despite the variable 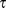, all land cover types exhibit some degree of change in fire selectivity as function of fire size. Such change is positive and its magnitude, described by the slope of the quantile regression, is higher in pine stands (0.288), shrublands (0.268), and eucalypt plantations (0.196), and lower in annual crops (0.074), thus suggesting that fire size has a stronger avoidance reducing effect in forest land cover types. Evergreen oak woodlands exhibit a distinct pattern, with avoidance increasing as fire size increases (−0.105).

Our results suggest that while small fires appear to be selective towards land cover, either through avoidance or preference, this effect fades as fires become larger. This agrees with previous work by [26], whom found that fire selectivity changes as a function of fire size. However, we observed that changes in selectivity are distinct according to the land cover type considered. Assuming that fire size is a proxy for severe meteorological conditions, we would expect to observe similar reduction in fire selectivity for all land cover types tested, however our results do not support this hypothesis. The effect of fire size on fire selectivity is clear for most forest types, but less pronounced for annual crops and evergreen oak woodlands. The estimated quantiles in the avoidance region show that selectivity of annual crops and oak woodlands is less affected by fire size than other land cover types. Low fire selectivity for evergreen oak woodlands and for rainfed cereal crops, mostly present in the southern half of Portugal, is likely to result from the low fuel loadings characteristic of these land use types. In northern Portugal, where population density is higher and settlement patterns are scattered, irrigated agriculture is more common, and low fire selectivity for croplands probably is due to a scarcity of available fuel, combined with more aggressive fire suppression in the extensive urban-cropland intermix. Moreover, it can be argued that during severe fire events, these areas remain as priorities in terms of fire suppression or eventually become more pressing, thus contributing for the relatively stable pattern of fire avoidance with increasing fire size [32].

In the ranking of increasing land cover proneness to fire, eucalypt stands rank between the least fire prone, annual crops, and the most fire prone, shrublands. In many parts of Portugal, eucalypts are cultivated in monospecific plantations for paper and pulp production, and such areas are under intensive fire management and protection. Due to their economic value and active management, fire incidence is generally lower, and suppression effectiveness in such areas may contribute to mitigate the effect of severe weather conditions.

Given the dynamic nature of fire drivers in the past decades in the western Mediterranean Basin [18], analysis of a longer time series would help characterize the significance of land cover as fire spread driver over time. Moreover, a greater understanding of the importance of fire regime drivers at national and local scale could be obtained if weather conditions were used as a predictor, instead of fire size. Comparing fire seasons occurring under variable weather conditions would allow for testing the hypothesis of a cause-effect relationship between fire weather and selectivity. Another important advantage of using weather data, rather than fire size, would be the ability to determine thresholds in meteorological variables that determine changes in selectivity, thus providing insight into meteorological conditions that act as turning points in fire behavior. For the same land cover type, fire behavior (and size) will vary depending on the characteristics of the surface fuels complex and local topography. This highlights the benefits of a regional analysis to identify the site-specific scale thresholds (e.g. thresholds in fire size) above which selectivity is altered. Such an analysis would also aid managers, by informing where fire drivers are more likely to be fuel-vs.weather- dominated, hence supporting preventive measures, such as fuel reduction or increasing vigilance and preparedness when severe fire weather is forecast. Additionally, including co-variates such as distance to roads or villages and landform position could be useful to fully understand what drives changes in selectivity for distinct land cover types.
